# One-pot method for preparing DNA, RNA, and protein for multiomics analysis

**DOI:** 10.1038/s42003-024-05993-1

**Published:** 2024-03-14

**Authors:** Stephanie Biedka, Duah Alkam, Charity L. Washam, Svitlana Yablonska, Aaron Storey, Stephanie D. Byrum, Jonathan S. Minden

**Affiliations:** 1 Impact Proteomics, LLC., Pittsburgh, PA 15206 USA; 2 Department of Biochemistry and Molecular Biology, University of Arkansas for Medical Sciences, Little Rock, AR 72205 USA; 3 Arkansas Children’s Research Institute, Little Rock, AR 72202 USA; 4 Department of Biomedical Informatics, University of Arkansas for Medical Sciences, Little Rock, AR 72205 USA

**Keywords:** Isolation, separation and purification, Cancer, Transcriptomics

## Abstract

Typical multiomics studies employ separate methods for DNA, RNA, and protein sample preparation, which is labor intensive, costly, and prone to sampling bias. We describe a method for preparing high-quality, sequencing-ready DNA and RNA, and either intact proteins or mass-spectrometry-ready peptides for whole proteome analysis from a single sample. This method utilizes a reversible protein tagging scheme to covalently link all proteins in a lysate to a bead-based matrix and nucleic acid precipitation and selective solubilization to yield separate pools of protein and nucleic acids. We demonstrate the utility of this method to compare the genomes, transcriptomes, and proteomes of four triple-negative breast cancer cell lines with different degrees of malignancy. These data show the involvement of both RNA and associated proteins, and protein-only dependent pathways that distinguish these cell lines. We also demonstrate the utility of this multiomics workflow for tissue analysis using mouse brain, liver, and lung tissue.

## Abstract (web summary)

The authors present a method utilizing the reversible protein tag ProMTag to prepare high-quality, sequencing-ready DNA and RNA, and either intact proteins or mass spectrometry-ready peptides for whole proteome analysis from a single sample.

## Introduction

The continuous development of high-throughput technologies has resulted in a wealth of biological data and new opportunities to understand complex biological systems. Multiomics data is becoming increasingly common and critical for the molecular characterization of many diseases^1–10^. No single ‘omics can provide comprehensive insight into disease^11,12^; for example, alterations to the genome or transcriptome do not necessarily translate to proteomic alterations^13^. Advances in multiomics data acquisition and accessibility are leading to the advent of precision medicine, as better understanding of the specific etiology of an individual’s disease will allow for more personalized and targeted treatment^14–17^.

A major challenge in multiomics research is the lack of standardization, including in sample preparation techniques^18–20^. Traditionally, multiomics studies that combine genomics, transcriptomics, and proteomics analyses rely on separate sample preparation workflows for each sample type^2,10,21–26^. Such split workflows tend to be laborious and expensive, relying on several separately purchased sample preparation kits or homemade protocols. Furthermore, dividing a tissue sample into several portions almost certainly introduces sampling artifacts due to tissue heterogeneity, giving rise to biased results. Commercially available kits that allow the collection of DNA, RNA, and protein from a single starting sample include the TriplePrep kit (Cytiva) and the AllPrep DNA/RNA/Protein kit (Qiagen). Neither of these kits yields mass spectrometry (MS)-ready peptides, requiring additional processing for any non-gel-based proteomics approach.

Here, we introduce a workflow to reproducibly produce high-quality DNA, RNA, and protein or peptides from a single sample for multiomics analysis. This workflow utilizes the bifunctional protein tag ProMTag^27^. One end of ProMTag forms a reversible, covalent link to protein primary amines (amino termini and lysine residues), while the other end can rapidly form an irreversible, covalent link using the click chemistry pair, methyltetrazine (MT) and trans-cyclooctene (TCO). We have shown that ProMTag allows for the covalent linkage of >90% of all proteins in a homogenate to a TCO-agarose bead matrix^27^. Because this linkage is stable in organic solvents, DNA and RNA can be co-precipitated with the protein coupled to the matrix. After washing away other cellular components, plus exogenously added salts and detergents, the RNA and DNA are sequentially resolubilized and collected. Finally, the protein is released from the TCO matrix by reversing the acid labile linkage between the ProMTag and the protein, yielding unmodified protein. The released protein can then be digested by the addition of an MT-modified trypsin (MT-Trypsin) that cleaves the proteins into peptides. MT-Trypsin also forms an irreversible covalent link to the TCO matrix, thus removing it from solution, yielding tryptic peptides ready for MS. This workflow generates three analysis-ready fractions of DNA, RNA, and peptides. Alternatively, intact protein that lacks all vestiges of the ProMTag can be collected by eliminating the trypsin digestion step.

To demonstrate the efficacy of this workflow, we analyzed four triple-negative breast cancer (TNBC) cell lines that exhibit different degrees of malignancy: MCF10A (non-tumorigenic), MCFNeoT (benign hyperplasia), MCFT1 (atypical hyperplasia), MCFCA1 (invasive cancer)^28,29^. TNBC is characterized by negative expression of estrogen, progesterone, and human epidermal growth factor receptor-2^30^. Compared to other breast cancer subtypes, TNBC tends to be more aggressive and invasive, and the prognosis for TNBC patients is usually poor, with mortality rates of 40% within 5 years of diagnosis^31^.

DNA, RNA, and peptides were prepared from each of these TNBC cell lines in triplicate and were analyzed by whole-genome sequencing (WGS), RNA sequencing (RNA-Seq), and MS, respectively. These data showed a high degree of reproducibility and agreed with published independent single-omics analyses.

An anticipated use of this multiomics workflow is the analysis of biopsies for more holistic molecular analysis. To demonstrate the efficacy of the workflow for tissue analysis, we prepared DNA, RNA, and peptides from as little as 1.6 mg of mouse liver, brain, and lung tissue. We show that these tissues yield sufficient amounts of sequencing-ready DNA and RNA, and MS-ready peptides.

## Results

### Preparation of DNA, RNA, and peptides via the ProMTag multiomics workflow

**Fig. 1:**
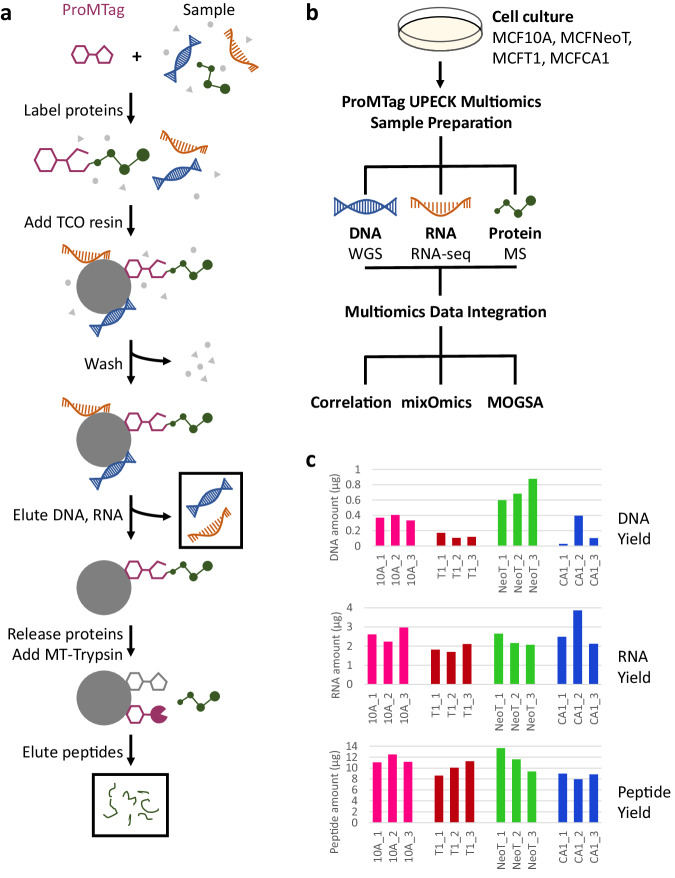
Preparation of DNA, RNA, and peptides from a single starting sample via the ProMTag Multiomics workflow. **a** The ProMTag Multiomics workflow. **b** TNBC cell lines were put through the ProMTag Multiomics workflow to produce DNA, RNA, and peptides that were analyzed via WGS, RNA-Seq, and mass spectrometry (MS), respectively. For each replicate, we began with a volume of cell lysate that contained 100 µg of protein. **c** Yields of DNA, RNA, and peptides from each replicate.

The ProMTag Multiomics workflow is outlined in Fig. 1a. First, ProMTag is added to a lysate and incubated at 4 °C for 30 minutes to label primary amines on proteins. The ProMTag-lysate is added to ProMTag Capture Resin; the MT moiety of the ProMTag reacts with this TCO resin, reversibly binding proteins to the resin during a 30-minute incubation at 4 °C. Nucleic acids are precipitated during this step by addition of acetonitrile. An initial series of wash steps remove contaminants without resolubilizing the nucleic acids. All wash steps are carried out in resin capture (RC) tubes, which have a small slit at the bottom of the tube. This slit allows liquid to pass through the RC-tube under centrifugal force or air pressure, while retaining the TCO resin along with the covalently linked proteins and precipitated DNA and RNA. RC-tubes have virtually zero void volume and eliminate the need for spin columns with polystyrene frits, which tend to bind proteins and peptides non-specifically. Each wash step throughout the workflow takes less than ten seconds.

The precipitated RNA and DNA are then eluted from the TCO resin agglomerate in two or three 5-minute elution steps. The composition of the first elution solution is designed to primarily resolubilize RNA, while the second elution solution yields the DNA fraction. Two elution steps are generally sufficient to recover most of the nucleic acids, but a third step can be added if the sample has large amounts of nucleic acids or if a more complete recovery is necessary. Since these fractions do not contain solely RNA or DNA, for this study, the first eluate was treated with DNase to yield pure RNA, while the second eluate was treated with RNase to yield pure DNA. The resulting RNA and DNA fractions were sufficiently pure for sequencing analysis without further cleanup.

To purify proteins and peptides that are covalently linked to the TCO resin, additional wash steps are done to remove any remaining nucleic acids. The proteins are then released from the TCO resin by the addition of an elution buffer, followed by a 15-minute incubation at room temperature. Released proteins are in their original, unmodified state^27^. MT-Trypsin is added to digest the proteins during a 1-hour incubation at 37 °C; this MT-Trypsin is captured on the TCO resin during digestion. Peptides are collected by centrifugation, and the resin is rinsed once in an elution buffer to recover any remaining peptides.

### Analysis of TNBC cell lines using the ProMTag multiomics workflow

To examine the efficacy and utility of the ProMTag Multiomics workflow, we compared four TNBC cell lines with differing degrees of aggressiveness: MCF10A (10A), MCFNeoT (NeoT), MCFT1 (T1), and MCFCA1 (CA1) (Fig. 1b). The 10A, NeoT, and T1 cell lines are pre-malignant while CA1 is an aggressive cancer cell line. These cell lines were grown to near confluence under standard conditions, washed to remove growth medium and dead cells, and lysed. Each lysate was subjected to the multiomics workflow in triplicate to test the repeatability of the method. The ProMTag Multiomics workflow generated sufficient quantity and quality of DNA, RNA, and peptides to perform WGS, RNA-Seq, and proteomic MS (Fig. 1c, Supplementary Fig. 2).

Across all samples, average yields were 0.35 µg DNA, 2.40 µg RNA, and 10.42 µg peptide. We observed some variability in DNA yield, with DNA amounts ranging from 0.03 µg to 0.88 µg (Fig. 1c). The lowest DNA yield sample, 0.03 µg of CA1_1 DNA, produced 3.3 × 10^8^ raw WGS reads and 99 G raw data, while the average raw reads and raw data across all samples were 3.2 × 10^8^ and 95.6 G, respectively. The number of WGS reads mapped ranged between 580 and 704 million reads with 100% mapping (Supplementary Fig. 1). Thus, the DNA yield from cell lysate containing 100 µg of protein was sufficient for WGS.

RNA yield across all samples ranged from 1.69 µg to 3.86 µg (Fig. 1c). This RNA was high quality, with RNA integrity numbers (RIN) of at least 8.0 (Supplementary Fig. 2). Half of the samples produced RNA with a RIN of >9. An average of 60 million RNA-Seq reads were mapped to the human genome resulting in quantitative analysis of 15,849 genes.

Likewise, the peptide yield across all samples was also sufficient for whole proteome analysis, with yields ranging between 8.0 and 13.6 µg of peptide per sample and an average yield of 10.4 µg protein per sample (Fig. 1c).

These data demonstrate that the ProMTag Multiomics workflow is capable of producing high-quality DNA, RNA, and peptides from a single sample using a one-pot cleanup method. A compelling attribute of our single-sample multiomics workflow is the ability to integrate WGS, RNA-Seq, and proteomics data without concern for sampling artifacts arising from ‘omics datasets obtained from separately isolated samples or tissue sections.

### Multiomics data analysis

**Fig. 2:**
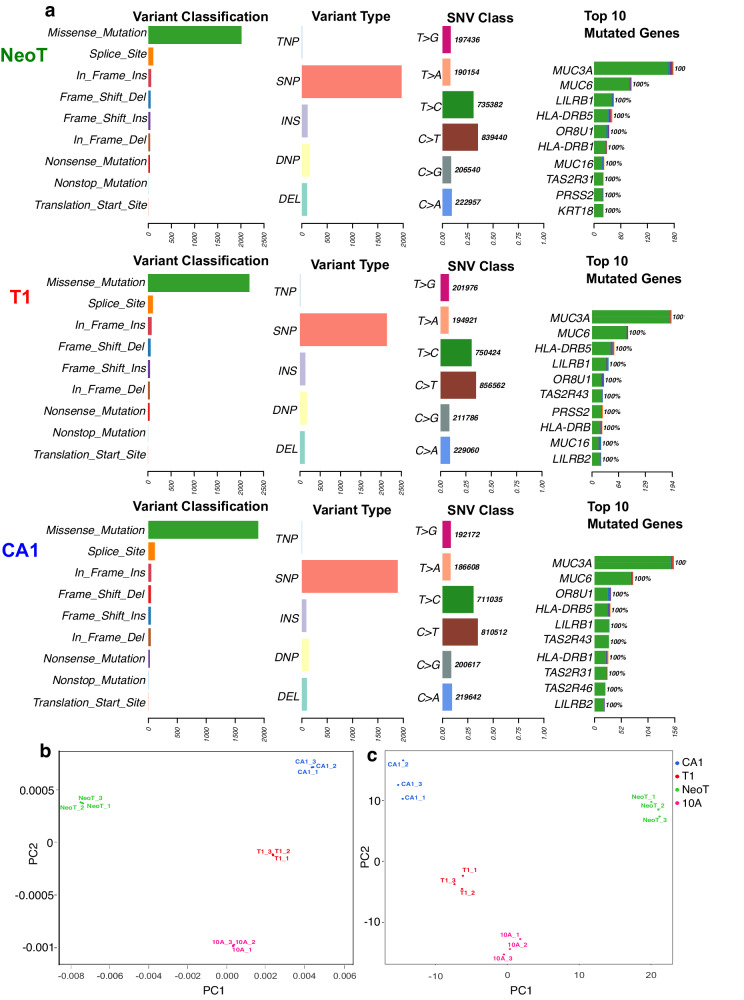
Quality of DNA, RNA, and protein data. **a** Summary of variant data detected using WGS. **b**, **c** PCA plots of transcriptomics and proteomics data, respectively.

WGS resulted in an average mapped reads of 617 million and a median coverage of 25× per sample (Supplementary Fig. 1). Mutect2 was used to identify variants in the NeoT, T1, and CA1 cell lines compared to the 10A control. A median of 2360, 2575, 2219 variants per sample were identified in NeoT, T1, and CA1, respectively (Fig. 2a). The majority of variants were classified as missense mutations with single nucleotide polymorphism (SNP) as the variant type. The top mutated genes include family members of Mucin (*MUC*) and *TAS2R*, which were significantly mis-regulated in the RNA-Seq datasets.

The RNA-Seq and proteomics samples generated high-quality datasets with a high degree of reproducibility, which is shown by the principal component analysis (PCA) (Fig. 2b, c). Each biological replicate in the RNA and protein data showed a nice degree of separation among the control and three cancer phenotypes with very little spread among the biological replicates within each group. PC1 explains 47% and 38% of the variability, while PC2 explains 27% and 25% for the RNA-Seq and proteomics data, respectively. Therefore, 74% of the variability in the RNA-Seq data and 63% of the variability in the MS data is accounted for within the first two components. These data indicate that the ProMTag Multiomics workflow is able to consistently and reproducibly extract DNA, RNA, and proteins from a single starting sample.

### Differential expression of RNA and protein

**Fig. 3:**
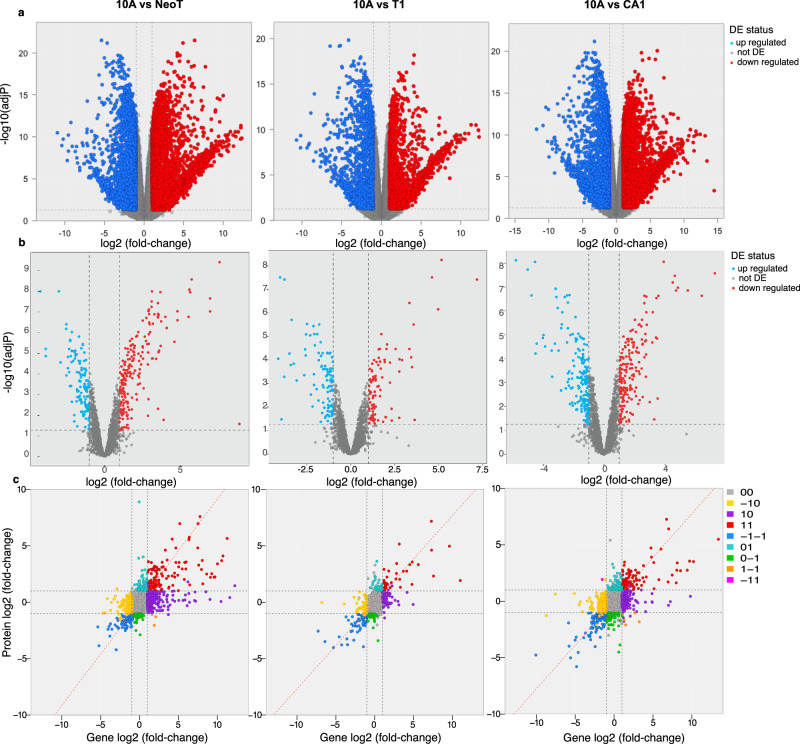
Differential expression of genes and proteins. **a**, **b** Volcano plots of transcriptomics and proteomics data, respectively. Differential expression (DE) status is color-coded to show significantly upregulated/downregulated molecules in red/blue. **c** Correlations between transcriptomics and proteomics data are shown.

The pre-malignant and cancerous TNBC cell lines were each compared to the 10A normal human mammary epithelial cells and analyzed for differential expression using RNA-Seq and proteomics. A total of 15,849 genes were quantified. Differential expression analysis resulted in 5638, 3107, and 5579 significant genes with a significance threshold of absolute fold change >2 and false discovery rate (FDR) adjusted *p* value < 0.05 when comparing 10A vs NeoT, T1, and CA1, respectively (Fig. 3a, Supplementary Data 1).

Eight of the *MUC* genes (*MUC1, MUC16, MUC5AC, MUC5B, MUC19, MUC21, MUC2*, and *MUC20*) were significantly different in at least one of the TNBC cell lines compared to the 10A. *MUC1* is overexpressed in ~90% of TNBCs and is a gene we would expect to see in our data set^32^. *MUC16* has also been identified as a top mutated gene in TNBCs^33,34^. *MUC16* has been shown to contribute to lung metastasis in TNBC patients, and depletion of *MUC16* results in decreased invasion, migration, and colony formation in TNBC cells^35^. Our RNA-Seq data shows *MUC1* and *MUC16* are both upregulated by log_2_ fold change 3.8 and 9.8 when comparing 10A vs NeoT, downregulated by log_2_ fold change (FC) of −0.3 and −3 when comparing 10A vs T1, and upregulated by log_2_ fold change of 8.4 and 0.54 when comparing 10A vs CA1 (Fig. 3a, Supplementary Data 1). *MUC1* and *MUC16* are significant in all comparisons with an FDR-adjusted *p* value < 0.05.

A total of 2926 proteins were quantified from triplicates of 10A, NeoT, T1, and CA1 cell lines using a data-independent acquisition (DIA) method (Fig. 3b, Supplementary Data 2). The number of significantly differentiated proteins with a significance threshold of absolute fold change >2 and FDR adjusted *p* value < 0.05 for the 10A vs NeoT, 10A vs T1, and 10A vs CA1 were 322, 200, and 383, respectively.

The Ensembl IDs from RNA were matched to the UniProt IDs in the proteomic data using the uniprot.org ID mapping tool. A total of 2837 genes and proteins were identified in both omics datasets and were correlated using Pearson correlation (Fig. 3c). The molecules identified as significantly differentiated with an FDR adjusted *p* value < 0.05 and an absolute fold change >2 (10A compared to TNBC) are color-coded on the correlation plot in Fig. 3c. Genes and proteins that were found upregulated are colored in red, while blue indicates down-regulation of both molecules. Interestingly, not all genes and proteins correlate in the same direction. These are indicated in the other quadrants in the correlation plot and are listed in Table 1 and Supplementary Data 3.

**Table 1:** The number of genes and proteins that are differentially expressed in the TNBC cell lines compared to 10A

Plot quadrant (gene/protein)	10A vs NeoT	10A vs T1	10A vs CA1
00 (not significant)	2065	2505	2083
10 (up in gene)	302	76	217
-10 (down in gene)	161	65	168
01 (up in protein)	69	49	91
0–1 (down in protein)	43	37	79
11 (up in both)	132	43	97
-1-1 (down in both)	63	60	95

Significance is defined as FDR *p* value <0.05 and absolute log_2_ fold change >1. A log_2_ fold change >1 indicates an upregulated molecule in 10A compared to the TNBC cell line. The colored dots in the correlation plot are indicated by the plot quadrant where 00 indicates both gene and protein are not significant, 01 indicates gene is not significant but the protein is significant and upregulated, while a 0–1 indicates gene is not significant but the protein is significant and downregulated.

**Fig. 4:**
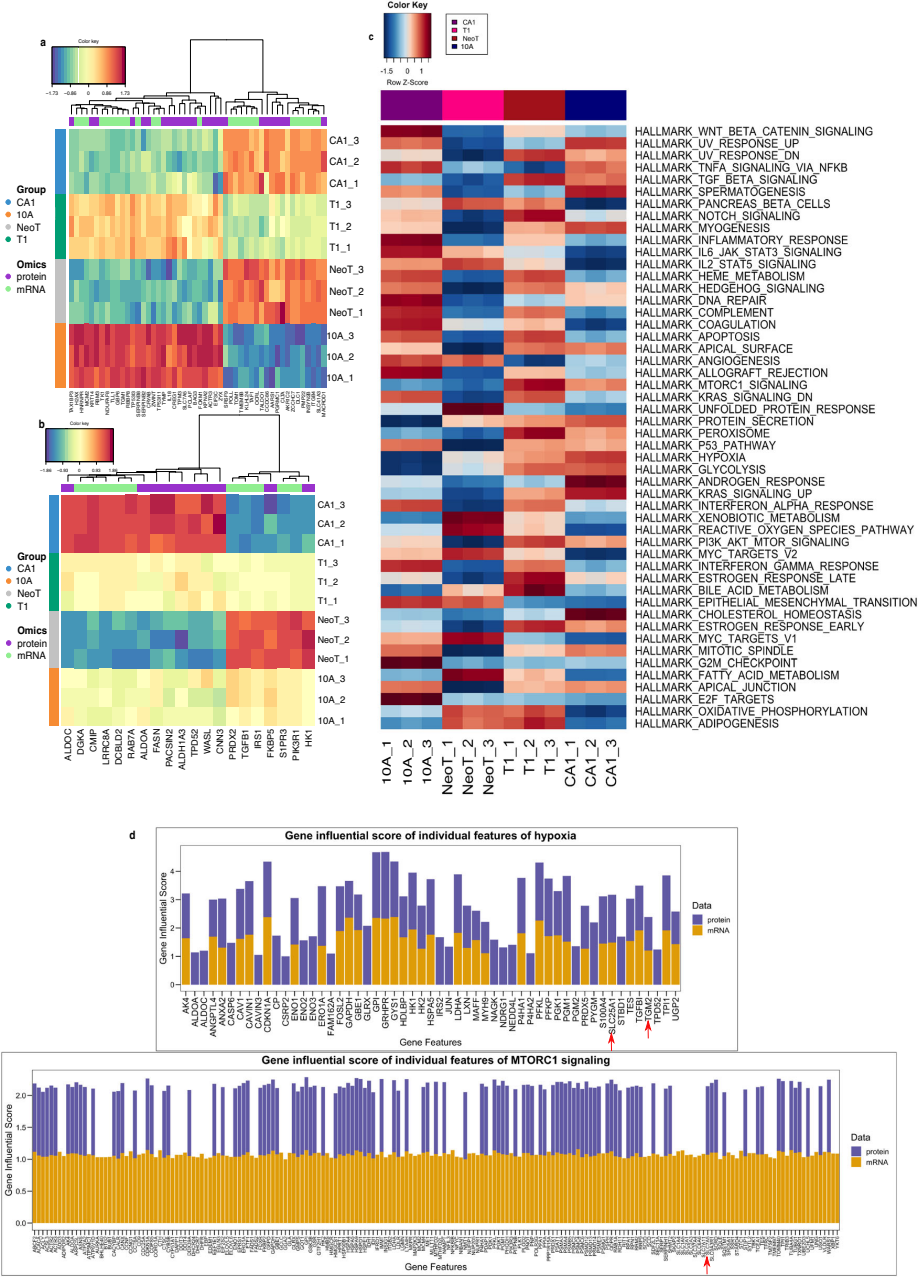
Integrated molecular features and pathways distinguishing the TNBC lines. The mixOmics R package was used to integrate the molecular features resulting from transcriptomics and proteomics analyses. The first (**a**) and second (**b**) principal components of the integration are shown. **c** The MOGSA R package was used to identify hallmark pathways significantly enriched or decreased as indicated by the transcriptomics and proteomics data. **d** Decomposition of the hypoxia (top) and mTOR (bottom) pathways to show the contribution of molecules within these pathways to the overall variance in the noted gene sets (PMID: 31243065).

Next, we used the mixOmics Data Integration Analysis for Biomarker discovery (DIABLO) method to extract molecular features from the RNA and protein datasets. The input uses all of the identified genes and proteins to provide a list of molecules separating all four cell lines using principal components. The top 50 molecules in the first and second principal components are shown in Figs. 4a, b. We then wanted to identify hallmark pathways and the contribution of RNA and protein defining these pathways in TNBC (Fig. 4c). Multiomics gene-set analysis (MOGSA) was used due to the fact the samples were prepared from the exact same biological sample. TNBC pathways such as TNF-α, p53, MTORC1 signaling, and TGF-β signaling pathways were all identified. Molecules such as *SLC7A5*, two of the TGM molecules (*TGM1*, *TGM2*), and several of the SLC molecules (*SLC41A3*, *SLC25A1*) were found in both the mixOmics (Fig. 4a, b) and MOGSA results (Fig. 4c). The contribution of the molecules from either the gene or protein level can be seen in the individual pathway bar charts in Fig. 4d. The more influence the molecule has for the pathway, the higher the gene influential score. The ProMTag Multiomics workflow successfully extracted DNA, RNA, and protein from each of the samples to perform multiomics level analyses and identified well-established pathways in TNBC.

### Preparation of DNA, RNA, and peptides from mouse tissues

**Fig. 5:**
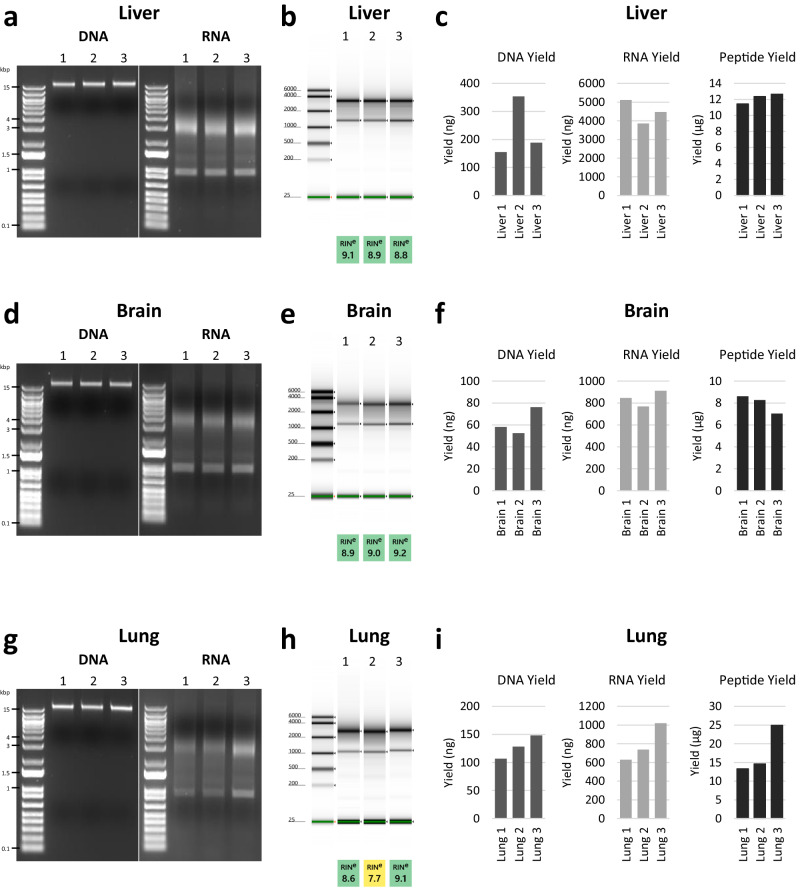
Preparation of RNA, DNA, and peptides from mouse tissues via the ProMTag multiomics workflow. The ProMTag Multiomics method was applied to three 5–7 mg pieces of mouse liver (**a**–**c**), brain (**d**–**f**), and lung (**g**–**i**). **a**, **d**, **g** Cleaned-up nucleic acids were run on 1% TAE agarose gels stained with ethidium bromide. **b**, **e**, **h** RINs were determined via a TapeStation RNA assay. **c**, **f**, **i** RNA and DNA yield were determined via Qubit dsDNA and RNA broad range assays, and peptide yield was assayed by a Pierce quantitative fluorometric peptide assay.

To show that this workflow is compatible with tissue samples as well as cultured cells, we prepared DNA, RNA, and peptides from mouse liver, brain, and lung tissue (Fig. 5). Liver, brain, and lungs were harvested from mice and pieces of each tissue were flash frozen. The ProMTag Multiomics workflow was then applied to these tissues with minor modifications to the previously described method. Specifically, we modified the composition of the lysis buffer and added a protein alkylation step immediately after nucleic acid elution. For tissues with high levels of RNase activity, RNase inhibitor was added to the first nucleic acid elution buffer.

A single 5–7 mg piece of tissue was used to prepare lysates from each tissue type. Since each tissue is composed of different cell types and different relative amounts of extra-cellular matrix, the protocol was altered to lyse tissue in a specific ratio of lysis buffer to tissue weight, 25 µL of lysis buffer per mg of tissue. Rather than measuring protein concentration prior to DNA, RNA, and protein isolation, 40 µL of tissue lysate was used per multiomics purification, which contains 1.6 mg of tissue. This generated a range of ~60 to 160 µg of input protein per sample (Supplementary Table 1). Here we analyzed tissue samples from three individual mice with three biological replicates for each tissue type. DNA, RNA, and peptide yield varied depending on the tissue type: from liver, an average of 233 ng of DNA, 4484 ng of RNA, and 12.2 µg of peptide was recovered, brain yielded an average of 62 ng DNA, 842 ng RNA, and 8.0 µg peptides, while lung provided an average of 128 ng DNA, 796 ng RNA, and 17.6 µg peptides (Fig. 5c, f, i, Supplementary Table 2). Nucleic acids were run on agarose gels (Fig. 5a, d, g), and RNA quality was also assessed via a TapeStation (Fig. 5b, e, h). The recovered RNA was high quality, with RIN ranging from 7.7 to 9.2, with only one sample producing a RIN under 8.6. While we did not carry out downstream analyses with these DNA, RNA, or peptides, the quality and yields are comparable to those obtained with the cultured TNBC cell lines (Fig. 1c, Supplementary Fig. 2). Together, these experiments demonstrate that the ProMTag Multiomics workflow is capable of generating high quality, analysis-ready DNA, RNA, and protein samples from tissue lysates.

## Discussion

Combining various ‘omics datasets into a single study is becoming increasingly important for researchers. However, until now these multiomics datasets were generated using different portions of a tissue specimen, which is prone to introducing sampling error or bias. For example, one portion of a specimen may have more cancer cells, while another portion may be more vascularized. In addition, each specimen portion is typically subjected to different ‘omics sample preparation methods, each with its own potential variability. Ideally, one would like to generate all multiomics data from the sample portion of tissue using a single workflow. Here, we introduce the ProMTag Multiomics workflow, which provides a novel solution to this problem, allowing the preparation of DNA, RNA, and protein in a single method, from a single starting sample.

This multiomics workflow depends on ProMTag, which is a novel, reversible, covalent protein tag that enables one to efficiently capture proteins on a solid support resin. This allows for the retention of proteins over a wide range of conditions, including organic solvents. We have taken advantage of this feature to precipitate nucleic acids along with protein capture, forming a DNA, RNA, protein agglomerate on the surface of the resin. Because of the different chemical attributes of these three macromolecules, we were able to produce three separate pools of DNA, RNA, and protein/peptides that were ready for downstream sequencing analysis. This workflow also relied on the use of RC-tubes which are fritless, zero void-volume, spin tubes for washing and eluting small volumes of capture resin. This method is highly reproducible and efficient, requiring about five hours of processing time.

Comparable multiomics sample preparation methods that are commercially available include the Qiagen AllPrep DNA/RNA/Protein kit. This kit relies on separate columns to purify DNA and RNA and yields intact proteins that must be precipitated before further downstream processing. Matthias Mann and collaborators have demonstrated that they were able to modify the AllPrep kit to allow for downstream MS of the AllPrep protein fraction^36^. In order to generate MS-ready peptides, they had to precipitate the proteins from the protein fraction, resuspend the proteins, digest, and cleanup the proteins using either the Protifi S-Trap™ kit or a traditional overnight trypsin digestion followed by desalting. With this approach, they were able to identify ~3300 protein groups per sample using data-dependent acquisition with an Easy-nLC Exploris setup. Importantly, they utilized 145-minute gradients for these samples, while the gradients for our ProMTag-prepared peptides were only 60 minutes. It is also important to point out that the protein count of 2926 reported here represented the fraction of proteins detected in at least nine of the twelve samples analyzed. The total number of proteins identified per individual sample is significantly higher. Furthermore, the AllPrep modifications described by Mundt et al.^36^ require protein precipitation and long trypsin digestion steps, while the ProMTag Multiomics method can be completed in ~5 hours. It is also worth noting that the Qiagen AllPrep DNA/RNA/Protein kit is not recommended for use with lipid tissues such as brain, whereas we were able to apply the ProMTag Multiomics approach to mouse brain samples successfully.

Here, we applied the ProMTag Multiomics workflow to four TNBC cell lines in triplicate. The resultant DNA, RNA, and peptide samples were analyzed by WGS, RNA-Seq, and MS, respectively. We observed strong correlation among replicates and between datasets, demonstrating the reproducibility of the ProMTag Multiomics workflow. Importantly, these datasets produced results that are in agreement with published studies. For example, we observed alterations in the expression of several of the *MUC* genes in the TNBC cell lines, and MOGSA identified pathways, including TNF-α, p53, MTORC1 signaling, and TGF-β signaling as hallmark pathways in the TNBC cell lines. This analysis highlights the importance of multiomics experiments, as we demonstrated that not all proteomic changes are observed at the DNA- or RNA-level. Thus, the combined multiomics approach is highly informative compared to any single ‘omics analysis.

We have demonstrated the efficacy of this method with both cultured cells and select mouse tissues. An important consideration when employing this method is extracting high-quality RNA from tissues that are particularly rich in RNases. During nucleic acid elution, the nucleic acids are in close proximity to captured proteins, including RNases, in a non-denaturing buffer. As such, there is a brief period during which RNA degradation can occur. This can be mitigated by the addition of an RNase inhibitor to the nucleic acid elution buffer. However, for RNase-rich tissues, such as spleen and pancreas, the amount of RNase inhibitor necessary to prevent RNA degradation might be unacceptable for downstream applications. This issue will need to be addressed in future applications.

Overall, the ProMTag Multiomics workflow is a unique method capable of producing DNA, RNA, and proteins of quality and quantity sufficient for downstream ‘omics applications from a small amount of a single starting sample. This method will help eliminate sampling bias and allow for more streamlined sample preparation for multiomics workflows. We anticipate this technology being of particular use in a medical setting, where samples are often limited and precious; the ability to carry out genomics, transcriptomics, and proteomics analyses of a single piece of biopsy tissue will improve our knowledge base, diagnosis, and treatment of diseases.

## Methods

### Cell lines and animals

MCF10A, MCFNeoT, MCFT1, and MCFCA1 cells were obtained from Olivera J. Finn (University of Pittsburgh). MCF10A cells were originally obtained from the American Type Culture Collection (ATCC; Manassas, VA, USA). MCFNeoT, MCFT1, and MCFCA1 cells were originally obtained from the Barbara Ann Karmanos Cancer Institute (Detroit, MI). Cells were maintained as monolayers in Dulbecco’s Modified Eagle’s Medium-F12 (DMEM/F12) (Gibco, 11320033) supplemented with 5% horse serum (Gibco, 16050122), 1% penicillin/streptomycin (Lonza, 17-602E), 0.5 μg/mL hydrocortisone (StemCell, 37150), 100 ng/mL cholera toxin (Sigma, C-8052), 10 μg/mL insulin (Gibco, 1285014), and 20 ng/mL recombinant human EGF (Invitrogen, PHG0311). All cell lines were regularly tested for Mycoplasma contamination by PCR.

To harvest cells for ProMTag Multiomics sample preparation, cells were washed four times with 5 mL ice-cold nuclease-free PBS (Sigma). Cells were lysed by addition of 1 mL 100 mM HEPES pH 8.0, 100 mM NaCl, 4 M guanidine thiocyanate. The lysate was scraped to a corner of the flask, and the flask was rinsed with an additional 250 µL 100 mM HEPES pH 8.0, 100 mM NaCl, 4 M guanidine thiocyanate. The lysate was flash-frozen and stored at −80 °C. Protein concentration was determined via a BCA assay (Pierce).

Mice used in this study were maintained in a Carnegie Mellon University animal facility following procedures conforming to the US NIH Guide for the Care and Use of Laboratory Animals^37^. Experiments were carried out in compliance with the Institutional Animal Care and Use Committee (IACUC) under approval numbers PROTO201600011 and PROTO201600045. Mice used in these experiments were healthy, untreated 2–3-month-old wild-type (C57B6) female mice. Mice were euthanized via isoflurane followed by cervical dislocation. Following animal sacrifice, tissues were promptly extracted, sliced into 2–4 mm pieces, and snap-frozen in liquid nitrogen. Frozen tissues were stored at −80 °C.

To prepare mouse tissue lysates for multiomics sample preparation, lysis buffer (100 mM HEPES pH 8.0, 100 mM NaCl, 4 M guanidine thiocyanate, 10 mM EDTA, 284 mM 2-mercaptoethanol) was added to a tissue piece at a ratio of 25 µL lysis buffer to 1 mg tissue. Tissue was homogenized on ice with an RNase-free disposable pellet pestle (Fisher Scientific) for 2-6 minutes until no tissue clumps were visible.

### Multiomics sample preparation via the ProMTag workflow

Cancer cell line multiomics sample preparation was carried out with the ProMTag Universal Protein Extraction and Cleanup Kit (UPECK)- Multiomics (Impact Proteomics). Proteins were labeled with ProMTag in buffer containing 100 mM HEPES pH 8.0, 100 mM NaCl, 4 M guanidine thiocyanate at a protein concentration of 2 mg/mL at 4 °C for 30 minutes.

Labeled lysate was added to 50 µL ProMTag capture resin in RC-tubes (Impact Proteomics), and 3 volumes of cold 100% acetonitrile (ACN) were added. The binding of labeled proteins to the capture resin was carried out at 4 °C with gentle rotation for 30 minutes.

The capture resin was washed 3 times with 200 µL 70% ACN. Ultrapure nuclease-free water (Invitrogen) was added to the capture resin to elute nucleic acids. Elution was carried out at 4 °C for 5 minutes with gentle rotation. The eluate was collected by brief centrifugation. A second nucleic acid elution step was carried out as described above using 10 mM Tris pH 8, 1 mM EDTA, 10 mM NaCl as the elution buffer.

After nucleic acid elution was completed, the capture resin was washed as follows: once with 200 µL 250 mM NaCl, 100 mM HEPES pH 8.0, 10% ACN, once with 200 µL 25% 100 mM HEPES pH 8.0, 75% ACN, and twice with 200 µL ultrapure water. Proteins were released from the capture resin by the addition of 100 mM formic acid (FA) followed by a 15-minute incubation at room temperature with gentle rotation. MT-Trypsin (Impact Proteomics) was added to the RC-tube and digestion was carried out at 37 °C for 1 hour. Peptides were recovered by briefly centrifuging the RC-tube. To recover any remaining peptides, 100 mM FA was added to the capture resin followed by a 5-minute incubation at room temperature with gentle rotation.

Mouse tissue multiomics samples were prepared as described above with the following modifications. For each sample, 40 µL tissue lysate in buffer containing 100 mM HEPES pH 8.0, 100 mM NaCl, 4 M guanidine thiocyanate, 10 mM EDTA, 284 mM 2-mercaptoethanol was labeled with 4.2 µL ProMTag. Labeled lysate was added to 100 µL ProMTag capture resin. For lung tissue samples only, the first nucleic acid elution buffer was supplemented with 2 units of 40 U/µL Protector RNase inhibitor (Roche) per 1 µL of elution buffer. Following nucleic acid elution, 50 µL 250 mM NaCl, 100 mM HEPES pH 8.0, 10% ACN, 20 mM iodoacetamide (IAA) was added to the ProMTag capture resin. The resin was incubated for 15 minutes at room temperature in the dark. Following protein alkylation, all steps were carried out as described above beginning with the wash with 200 µL 250 mM NaCl, 100 mM HEPES pH 8.0, 10% ACN.

### DNA sample preparation and whole-genome sequencing (WGS)

Nucleic acid concentration in nucleic acid eluates was determined via Qubit dsDNA BR and Qubit RNA BR (Invitrogen) assays. If the amount of DNA in the second nucleic acid eluate was insufficient for downstream applications, a fraction of the first eluate was transferred to the second eluate. To prepare DNA for whole-genome sequencing, 1.0 µL of 4 mg/mL RNase A solution (Promega) was added to the DNA followed by a 30-minute incubation at 37 °C. DNA samples were stored at −80 °C.

WGS was performed by Novogene. Briefly, the genomic DNA was sheared into 350 bp fragments, libraries were constructed using the NEBNext DNA Library Prep Kit, followed by end repair, dA-tailing, and ligation with NEBNext adapter. Fragments (300-500 bp) were PCR enriched by P5 and indexed P7 oligos. DNA libraries were checked for quantity and quality using Qubit 2.0 fluorometer and the Agilent 2100 bioanalyzer, respectively. The Illumina Novaseq 6000 (Illumina Inc., San Diego, CA, USA) was utilized for WGS in Novogene Bioinformatics Technology Co., Ltd (Beijing, China) to generate 150 bp paired-end reads.

### WGS analysis

DNA sequencing files were processed using the nf-core/sarek pipeline (v3.0.1)^38^. The human genome GRCh38 was used as the reference genome. The Mutect2 tool offered by sarek v3.0.1 was applied to detect variants, the snpEff tool was used to annotate the variants. Variant summary plots were generated using Maftools^39^.

### RNA sample preparation and mRNA sequencing

To prepare RNA for mRNA sequencing, 10× DNase I buffer (Ambion) was added to a final concentration of 1× and 1.0 µL of 2 U/µL RNase-free DNase I (Ambion) was added. The reaction was incubated at 37 °C for 30 minutes. EDTA was added to a final concentration of 5 mM, and the RNA was heated at 75 °C for 10 minutes to inactivate DNase I. RNA samples were stored at −80 °C.

For mouse tissues, DNA and RNA were treated with RNase and DNase, respectively. RNase treatment was carried out as described above. DNase treatment was carried out at room temperature (~21 °C) for 30 minutes (liver and brain) or 5 minutes (lung). Nucleic acids were then loaded on a 1% TAE (Thermo Scientific) agarose gel stained with ethidium bromide (Invitrogen) and electrophoresed for 30 minutes at 120 V. To assess the quantity of mouse tissue RNA, RNA concentration was measured with the Qubit™ RNA Broad Range assay kit (ThermoFisher). RNA samples were diluted with nuclease-free water to an appropriate concentration. RINs were determined using a High Sensitivity RNA ScreenTape assay (Agilent) on an Agilent 4150 TapeStation following the manufacturer’s protocol and using an electronic ladder. The RINs were analyzed in the TapeStation Controller software.

A total of 1 µg RNA per sample was used to generate sequencing libraries using the NEBNext Ultra RNA Library Prep kit for Illumina (NEB, USA) following manufacturer’s recommendations. RNA purity was checked using the NanoPhotometer spectrophotometer (IMPLEN, CA, USA). RNA integrity and quantitation were assessed using the RNA Nano 6000 Assay kit of the Agilent 2100 Bioanalyzer. Sequencing was performed by Novogene Bioinformatics Technology on an Illumina NovaSeq 6000 platform with paired-end sequencing.

RNA-Seq data was analyzed using the nf-core/rnaseq pipeline (v3.4; doi: 10.5281/zenodo.1400710)^40^. The human genome GRCh38 was used as the reference genome. Counts obtained using the ‘--aligner_star_salmon’ option were transformed to log_2_ counts per million (CPM)^41^. Further details of RNA-Seq data analysis are in the Statistics and Reproducibility section below.

### Preparation of peptide samples and mass spectrometry analysis

To determine peptide yield, 15 µL of peptide eluate was transferred to a fresh tube. This fraction and the remainder of the peptide eluate were dried fully in a SpeedVac system (Thermo Scientific Savant). The 15 µL fraction was resuspended in 15 µL 100 mM HEPES, and peptide yield was determined via a Quantitative Fluorometric Peptide Assay (Pierce). Dried peptide samples were stored at −80 °C.

1 µg of tryptic peptides from each sample were separated by reverse phase XSelect CSH C18 2.5 um resin (Waters) on an in-line 150 × 0.075 mm column using an UltiMate 3000 RSLCnano system (Thermo Scientific). Peptides were eluted using a 60 min gradient from 98:2 to 65:35 buffer A:B ratio (Buffer A = 0.1% FA, 0.5% ACN; Buffer B = 0.1% FA, 99.9% ACN). Eluted peptides were ionized by electrospray (2.2 kV) followed by mass spectrometric analysis on an Orbitrap Exploris 480 mass spectrometer (Thermo Scientific). To assemble a chromatogram library, six gas-phase fractions were acquired on the Orbitrap Exploris with 4 m/z DIA spectra (4 m/z precursor isolation windows at 30,000 resolution, normalized AGC target 100%, maximum inject time 66 ms) using a staggered window pattern from narrow mass ranges using optimized window placements. Precursor spectra were acquired after each DIA duty cycle, spanning the m/z range of the gas-phase fraction (i.e., 496–602 m/z, 60,000 resolution, normalized AGC target 100%, maximum injection time 50 ms). For wide-window acquisitions, the Orbitrap Exploris was configured to acquire a precursor scan (385–1015 m/z, 60,000 resolution, normalized AGC target 100%, maximum injection time 50 ms) followed by 50 × 12 m/z DIA spectra (12 m/z precursor isolation windows at 15,000 resolution, normalized AGC target 100%, maximum injection time 33 ms) using a staggered window pattern with optimized window placements. Precursor spectra were acquired after each DIA duty cycle.

Following data acquisition, data were searched using an empirically corrected library against the UniProt *Homo sapiens* database (August 2022) without the modification for carbamidomethylation since IAA was not used during the sample preparation. Quantitative analysis was performed to obtain a comprehensive proteomic profile. Proteins were identified and quantified using EncyclopeDIA (version 1.12.31)^42^ and visualized with Scaffold DIA (version 3.3.1). Further details of MS data analysis are in the Statistics and Reproducibility section below.

### Data integration

RNA-Seq and proteomics data were normalized independently to account for technique-specific variability and limitations. The methods of normalization are described above for each specific ‘omics technique. The normalized datasets with consistent annotation were integrated using techniques such as Pearson correlation analysis and matrix factorization using the tools mixOmics and MOGSA.

Multiomics data annotation was mapped based on the Ensembl ID obtained from uniprot.org in order to match the RNA-Seq and proteomics datasets. The *Homo sapiens* UniProt ID mapping for UniProt_Release_2023_01 was used to map Ensembl IDs to the protein data set. In total both RNA and protein datasets contained 2839 Ensembl IDs in common. Pearson correlation analysis was performed using the significant features from each data set, which corresponds to the four outer quadrants in Fig. 3c, to identify the relationships between DNA methylated promoters and the genes they regulate, between gene and protein expression, and between protein expression and phosphorylated proteins.

The gene and protein normalized expression data was analyzed using mixOmics in order to identify molecular features associated with the cancer phenotype^43^. mixOmics applies matrix factorization using the supervised projection to latent structures models for data integration to reduce the dimension, capture and explain the variation in the data that discriminate between 10A, NeoT, T1, and CA1 cell lines. We used DIABLO with the sparse partial least squares regression method with tuning parameter set to keep the top 50 features.

Multiomics gene-set analysis (MOGSA) was also applied to identify Hallmarks of cancer pathways. MOGSA is a multivariate single-sample gene-set analysis method that integrates multiple experimental and molecular data types measured over the same set of samples^44^. The method learns a low dimensional representation of most variant correlated features (genes and proteins), transforms the features onto the same scale, and calculates an integrated gene-set score (GSS) from the most informative features in each data type. A gene set with a high GSS is driven by features that explain a large proportion of the global correlated information among data matrices and can be from one or all data matrices.

### Statistics and reproducibility

All TNBC cell line multiomics experiments were carried out in technical triplicates, and all mouse multiomics experiments were carried out in biological triplicates.

For TNBC RNA-Seq data, low-expressed genes were filtered out, and libraries normalized by the trimmed mean of M-values^45^. Differential expression was performed using linear models for microarray data (limma) voom with quality weights, and *p* values were corrected for multiple testing using the Benjamini-Hochberg procedure^46^. Genes with a FDR *p* value < 0.05 and fold change >2 were considered significant.

For TNBC MS data, proteins were identified and quantified using EncyclopeDIA (version 1.12.31)^42^ and visualized with Scaffold DIA (version 3.3.1) using 1% false discovery thresholds at both the protein and peptide levels. For each peptide, the 8 highest quality fragment ions were selected for quantitation. Protein-exclusive intensity values were assessed for quality using ProteiNorm^47^. The data was normalized using variance stabilizing normalization (VSN)^48^ and statistical analysis was performed using limma with empirical Bayes (eBayes) smoothing to the standard errors^46^. Proteins with an FDR adjusted *p* value < 0.05 and a fold change >2 were considered significant.

### Reporting summary

Further information on research design is available in the Nature Portfolio Reporting Summary linked to this article.

### Supplementary information

Supplementary Information

Description of Additional Supplementary Files

Supplementary Data 1

Supplementary Data 2

Supplementary Data 3

Reporting Summary

## Data Availability

Whole-genome sequencing and RNA-Seq data have been uploaded to the GEO repository (Reference Number GSE241434). Proteomics data have been submitted to the MassIVE repository (Reference Number MSV000092780). Unedited gel images for cropped gels shown in Fig. 5 are available in Supplementary Fig. 3. All other data are available upon request from Dr. Jonathan Minden.
