# Supplementary material for: One-pot method for preparing DNA, RNA, and protein for multiomics analysis
Source: Commun Biol. 2024 Mar 14;7:324. doi: 10.1038/s42003-024-05993-1 (PMC10940598; doi:10.1038/s42003-024-05993-1)
Supplement: Supplementary file 1 — Supplementary Information [file 42003_2024_5993_MOESM1_ESM.pdf]

Supplementary Information

One-pot method for preparing DNA, RNA, and protein for multiomics analysis

Stephanie Biedka[1], Duah Alkam[2], Charity L. Washam[2], Svitlana Yablonska[1], Aaron Storey[2], Stephanie D. Byrum[2,3,4], Jonathan S. Minden[1,5]

[1]Impact Proteomics, LLC., Pittsburgh, PA 15206, USA

[2]Department of Biochemistry and Molecular Biology, University of Arkansas for Medical Sciences, Little Rock, AR 72205, USA

[3]Arkansas Children's Research Institute, 13 Children's Way, Little Rock, AR 72202, USA

[4]Department of Biomedical Informatics, University of Arkansas for Medical Sciences, Little Rock, AR 72205, USA

[5]Corresponding author. Email address: jonathan.minden@impactproteomics.com

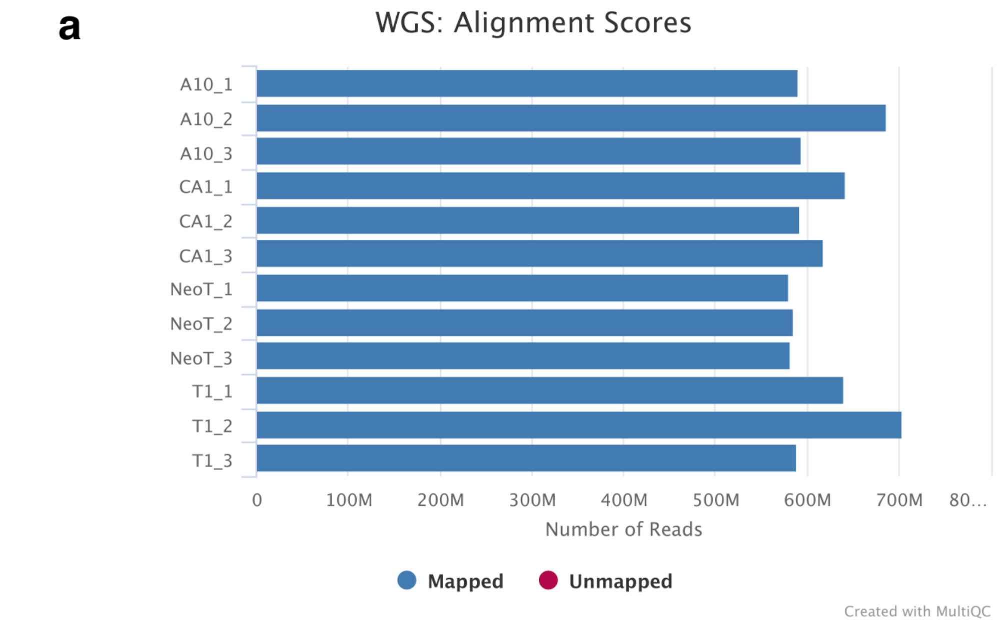

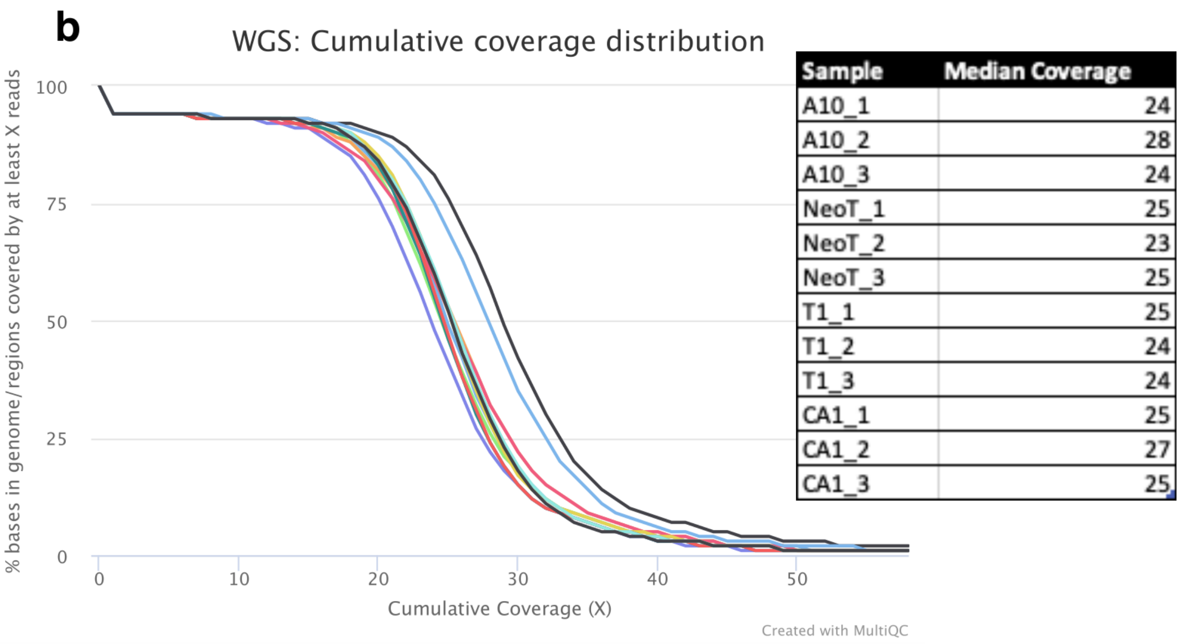

Sample	Median Coverage
A10_1	24
A10_2	28
A10_3	24
NeoT_1	25
NeoT_2	23
NeoT_3	25
T1_1	25
T1_2	24
T1_3	24
CA1_1	25
CA1_2	27
CA1_3	25

Supplementary Figure 1. Quality of WGS data. **(a)** WGS sequencing reads aligned to the human reference genome at 100% in all libraries with median coverages of 24-28 **(b)**.

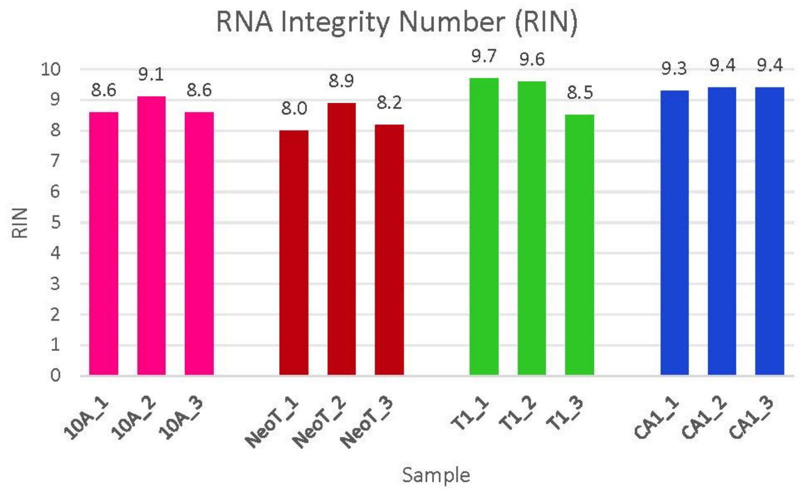

Supplementary Figure 2. RNA integrity numbers (RINs) for RNA prepared from each TNBC sample via the ProMTag Multiomics workflow.

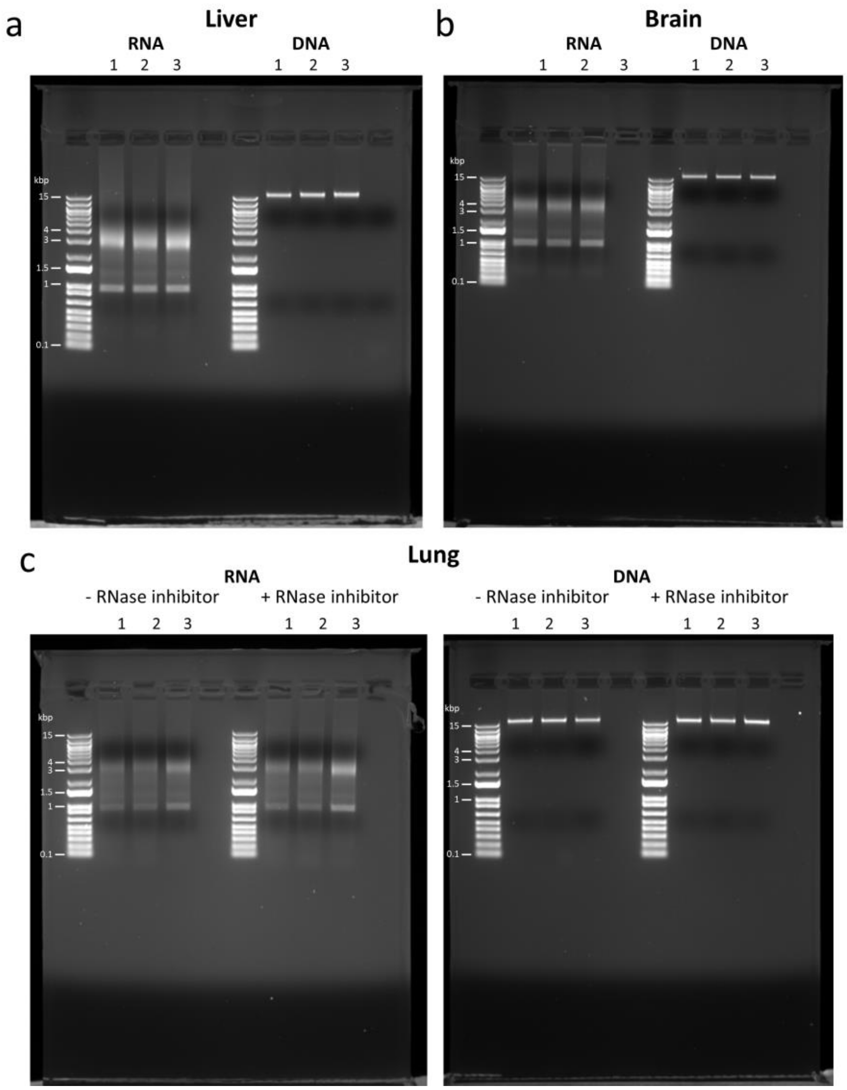

Supplementary Figure 3. Unedited gel images for cropped gels shown in Figure 5. **(a)** Unedited gel shown in Figure 5a. **(b)** Unedited gel shown in Figure 5d. **(c)** Unedited gels shown in Figure 5g. Note that the + RNase inhibitor samples from both gels are shown as a single composite gel in Figure 5g.

Tissue sample	Protein input (µg)	Tissue weight (mg)
Liver 1	62.8	7.6
Liver 2	75.7	5.4
Liver 3	66.3	7.3
Brain 1	57.8	7.5
Brain 2	65.6	9.7
Brain 3	57.8	8.8
Lung 1	148.8	5.1
Lung 2	154.4	4.8
Lung 3	102.4	5.9

Supplementary Table 1. Protein input for mouse tissue ProMTag Multiomics sample preparations.

Tissue sample	RNA conc. (ng/µl)	RNA yield (ng)	**Average RNA yield (ng)**	DNA conc. (ng/µl)	DNA yield, ng	**Average DNA yield (ng)**	Peptide conc. (µg/mL)	Peptide yield (µg)	**Average peptide yield (µg)**
Liver 1	85.3	5118		2.6	155		114.5	11.4	
Liver 2	64.3	3858	**4484**	5.9	354	**233**	123.7	12.4	**12.2**
Liver 3	74.6	4476		3.1	188		126.7	12.7	
Brain 1	14.1	846		1.0	58		86.2	8.6	
Brain 2	12.8	768	**842**	0.9	53	**62**	82.7	8.3	**8.0**
Brain 3	15.2	912		1.3	76		70.3	7.0	
Lung 1	10.5	630		1.8	107		133.0	13.3	
Lung 2	12.3	738	**796**	2.1	128	**128**	146.1	14.6	**17.6**
Lung 3	17.0	1020		2.5	148		249.4	24.9	

Supplementary Table 2. DNA, RNA, and peptide yield from mouse liver, brain, and lung prepared via the ProMTag Multiomics workflow.

Supplementary Data 1, 2, and 3 are provided separately.

Supplementary Data 1. RNA-Seq analysis. The normalized counts for each sample are included along with the limma voom statistical results for each comparison.

Supplementary Data 2. Proteomics data. The VSN normalized MS1 protein intensities for each sample is provided along with the limma statistical output for each comparison.

Supplementary Data 3. Correlation of the RNA-Seq and proteomics data sets. Plots are shown in Figure 3c.
