# Supplementary material for: One-pot method for preparing DNA, RNA, and protein for multiomics analysis
Source: Commun Biol. 2024 Mar 14;7:324. doi: 10.1038/s42003-024-05993-1 (PMC10940598; doi:10.1038/s42003-024-05993-1)
Supplement: Supplementary file 2 — Description of Additional Supplementary Files [file 42003_2024_5993_MOESM2_ESM.pdf]

Description of Additional Supplementary Files

File name: Supplementary Data 1
Description: RNA-Seq analysis. The normalized counts for each sample are included along with the limma voom statistical results for each comparison.

File name: Supplementary Data 2
Description: Proteomics data. The VSN normalized MS1 protein intensities for each sample is provided along with the limma statistical output for each comparison.

File name: Supplementary Data 3
Description: Correlation of the RNA-Seq and proteomics data sets. Plots are shown in Figure 3c.
